# Transgender and other gender diverse adolescents with eating disorders requiring medical stabilization

**DOI:** 10.1186/s40337-022-00722-7

**Published:** 2022-12-23

**Authors:** Anita V. Chaphekar, Stanley R. Vance, Andrea K. Garber, Sara Buckelew, Kyle T. Ganson, Amanda Downey, Jason M. Nagata

**Affiliations:** 1 Department of Pediatrics, University of California, San Francisco, 550 16th Street, Box 0503, San Francisco, CA 94143 USA; 2 Factor-Inwentash Faculty of Social Work, University of Toronto, 246 Bloor St W, Toronto, ON Canada; 3 Department of Psychiatry and Behavioral Sciences, University of California, San Francisco, 550 16th Street, Box 0503, San Francisco, CA 94143 USA

**Keywords:** Transgender, Gender diverse, Non-binary, Gender identity, Eating disorder, Inpatient medical unit, Treatment goal weight, Percent median body mass index, Growth chart, Adolescents

## Abstract

**Background:**

Despite the high prevalence of eating disorders in gender diverse adolescents, little is known about the characteristics of gender diverse youth with eating disorders who require inpatient medical stabilization. The primary objective of this study was to describe the medical, anthropometric, and psychiatric characteristics of gender diverse adolescents hospitalized for eating disorders and compare these characteristics to cisgender peers hospitalized for eating disorders. The secondary objective was to evaluate percent median body mass index as one marker of malnutrition and treatment goal body mass index as a recovery metric between patients’ birth-assigned sex and affirmed gender using standardized clinical growth charts.

**Methods:**

A retrospective chart review was conducted of 463 patients admitted to an inpatient eating disorders medical unit between 2012 and 2020. To compare medical, anthropometric, and psychiatric data between gender diverse and cisgender patients, chi-square/Fisher’s exact and *t*-tests were used. Clinical growth charts matching the patients’ birth-assigned sex and affirmed gender identity were used to assess percent of median body mass index and treatment goal body mass index.

**Results:**

Ten patients (2.2%) identified as gender diverse and were younger than cisgender patients [13.6 (1.5) years vs. 15.6 (2.7) years, *p* = 0.017]. Gender diverse patients were hospitalized with a higher percent median body mass index compared to cisgender peers [97.1% (14.8) vs. 87.9% (13.7), *p* = 0.037], yet demonstrated equally severe vital sign instability such as bradycardia [44 (8.8) beats per minute vs. 46 (10.6) beats per minute, *p* = 0.501], systolic hypotension [84 (7.1) mmHg vs. 84 (9.7) mmHg, *p* = 0.995], and diastolic hypotension [46 (5.8) mmHg vs. 45 (7.3) mmHg, *p* = 0.884]. Gender diverse patients had a higher prevalence of reported anxiety symptoms compared to cisgender patients (60% vs. 28%, *p* = 0.037).

**Conclusions:**

Gender diverse patients demonstrated complications of malnutrition including vital sign instability despite presenting with a higher weight. This is consistent with a greater proportion of gender diverse patients diagnosed with atypical anorexia nervosa compared to cisgender peers. Additionally, psychiatric comorbidities were present among both groups, with a larger percentage of gender diverse patients endorsing anxiety compared to cisgender patients.

## Plain English Summary

Gender diverse is a term that includes gender identities such as (but not limited to) transgender, non-binary, and gender fluid. Eating disorders are becoming increasingly recognized among this population. In recent years there has been a better understanding of the risk factors and screening for eating disorders in the outpatient setting for gender diverse adolescents. However, the medical, anthropometric, and psychiatric characteristics of gender diverse adolescents with eating disorders requiring inpatient medical stabilization are poorly understood. This study examines these characteristics in gender diverse adolescents hospitalized for eating disorder-related medical complications from 2012 to 2020. Gender diverse adolescents presented for medical hospitalization with signs of malnutrition, including vital sign instability and laboratory abnormalities, as well as additional psychiatric diagnoses such as depression and anxiety. Understanding the medical, anthropometric, and psychiatric presentation of this population can lead to more tailored and comprehensive care with the potential to reduce the risks of adverse health outcomes.

## Background

Gender diverse individuals experience an incongruity between their sex assigned at birth and their gender identity [1–3]. While the terminology for this population is varied, we will use the terms ‘transgender male’/male gender identity to refer to a birth-assigned female who identifies as male, ‘transgender female’/female gender identity to refer to a birth-assigned male who identifies as female, ‘non-binary’ to refer to individuals who do not identify as either male or female, ‘gender fluid’ to refer to individuals whose gender identity is not fixed, ‘cisgender’ to refer to individuals whose gender identity aligns with their birth-assigned sex, and ‘gender diverse’ as an overarching term to encompass the diversity of gender identities other than cisgender [3–5]. We define gender dysphoria as distress related to the incongruence between a person’s birth-assigned sex and gender identity [6]. Additionally, we define body dissatisfaction as negative feelings toward one’s body which typically takes on a key role in gender dysphoria [1].

Transgender and other gender diverse individuals are at increased risk for development of eating disorders due to significant risk factors including stigma, discrimination, and body dissatisfaction [7–10]. This population may engage in eating disorder behaviors to mitigate or suppress secondary sex characteristics [1, 7, 10–15]. For example, the desire to suppress menstruation and other secondary female sex characteristics are risk factors for disordered eating behaviors in transgender males [2, 16]. Similarly, the desire for ‘thinness’ or to minimize secondary male sex characteristics are associated with eating disorder behaviors in transgender females [2, 17]. Striving for perfectionism, anxiety symptoms, and low self-esteem are additional risk factors for eating disorders among transgender individuals [2, 18].

Gender diverse individuals experience disproportionately high rates of mood disorders, self-injurious behaviors, and suicidality which are associated with gender dysphoria, bullying, victimization, and other forms of stigmatization [19–22]. Notably, transgender and gender diverse individuals with eating disorders have the highest rates of self-harm, suicidal ideation, and suicide attempts [7, 10]. As psychiatric comorbidities often portend negative long-term outcomes in those with eating disorders, it is critical to examine gender diverse adolescents who experience eating disorders to better understand their unique risk and vulnerability for psychiatric comorbidities and medical instability [23–27].

Risk factors and symptomatology of gender diverse adolescents seeking outpatient care for eating disorders has recently been explored [14]. However, a gap remains in the presenting medical, anthropometric, and psychiatric characteristics of gender diverse adolescents with eating disorders that require medical hospitalization. As medical hospitalizations for eating disorders continue to rise, it is imperative to understand these characteristics of gender diverse adolescents in order to deliver tailored, evidence-based interventions for this vulnerable population [28, 29].

Percent median body mass index (% mBMI) is one widely used marker to determine the degree of malnutrition with less than 70% mBMI indicating severe malnutrition, 70–79% mBMI indicating moderate malnutrition, and 80–90% mBMI indicating mild malnutrition [30]. This metric is often used to determine if a patient requires an inpatient level of care.

Treatment goal weight (TGW) represents a weight in which a patient demonstrates medical and psychologic recovery from an eating disorder [31]. TGW is determined by a variety of factors including the patient’s age, height, previous growth trajectory, and pubertal stage [31, 32]. As such, the TGW should be individualized with each patient [31]. TGW is an important metric in the care of individuals hospitalized for eating disorders as TGW is used to calculate the caloric goal of refeeding protocols which has been shown to affect the length of hospital stay [33]. In other words, patients with higher TGW will have higher caloric goals. When using an evidence-based refeeding approach which starts at a caloric threshold and advances systematically day by day, it will take longer to reach a higher caloric goal and lengthen hospital stays [33]. For the purposes of our study, we will be using treatment goal body mass index (BMI) which is determined similarly to TGW.

Standard clinical practice uses sex-based growth charts to establish weight trajectory targets for the medical and psychological recovery of young people with eating disorders. This practice can present a clinical challenge for providers caring for individuals treated with gonadotropin-releasing hormone (GnRH) agonist therapy as a form of pubertal suppression or for those utilizing feminizing or masculinizing gender affirming medications [2, 7, 34, 35]. The current recommendations for establishing TGW for gender diverse adolescents on affirming therapy are for providers to refer to growth charts for the individual’s birth-assigned sex and gender identity [2, 34, 35]. Kidd et al. has previously demonstrated this using theoretical BMI measures, however, to our knowledge, this has not been demonstrated with real-world patient anthropometric measures [34]. Research informing the use of growth charts in the gender diverse population will allow for the provision of individualized and gender-affirming treatment.

The purpose of this study is to expand the knowledge of gender diverse adolescents hospitalized for medical complications of eating disorders. We describe the medical, anthropometric, and psychiatric characteristics in 10 gender diverse adolescents admitted to a large inpatient medical stabilization unit between 2012 and 2020 and compare these characteristics to cisgender patients. We also compare % mBMI as a marker of nutritional status and treatment goal BMI as a recovery metric between patients’ birth-assigned sex growth chart and gender-affirmed clinical growth chart for those with a binary gender identity and birth-assigned sex growth chart with ‘alternative’ growth chart for those with a non-binary identity.

## Methods

Ten gender diverse adolescents were identified through retrospective chart review of 463 adolescents and young adults, aged 9–25 years, admitted to a large inpatient medical stabilization unit at the University of California, San Francisco between May 2012 and August 2020. Patients were considered gender diverse if their birth-assigned sex was different from their gender identity as listed in the history and physical at time of admission. Patients were considered cisgender if their birth-assigned sex was the same as their listed gender identity. Patients were also categorized as cisgender if they did not have gender identity listed at time of hospitalization. Gender identity was categorized as transgender male/male gender identity, transgender female/female gender identity, non-binary, and other. Other included gender fluid and ‘no label’.

Demographic data collected included age, birth-assigned sex, gender identity, race, and ethnicity. Medical and anthropometric data collected included weight at time of admission, height, vital signs during hospitalization, treatment goal BMI from clinical notes, laboratory analysis, and utilization of gender-affirming medical therapy. Psychiatric data collected included eating disorder diagnoses and comorbid psychiatric diagnoses.

Patients were admitted with eating disorder diagnoses already known (as determined by outpatient health care providers) or were newly diagnosed after psychological assessment during hospitalization. Eating disorder diagnoses were reclassified per Diagnostic and Statistical Manual of Mental Disorders, Fifth Edition (DSM-5) criteria for those patients diagnosed using Diagnostic and Statistical Manual of Mental Disorders, Fourth Edition (DSM-IV) criteria [6]. To allow for sufficient sample for analyses, eating disorder diagnosis was classified into three categories: anorexia nervosa (AN), other specified feeding and eating disorders (OSFED) which includes atypical anorexia nervosa, and other. ‘Other’ included avoidant restrictive food intake disorder, bulimia nervosa, and unspecified eating disorder.

Psychiatric diagnoses for patients included self-reported pre-existing psychiatric diagnoses and psychiatric diagnoses made during hospitalization by psychologists. We created a category of depression which included the DSM-IV and DSM-5 diagnoses of major depressive disorder, depressive disorder not otherwise specified, and unspecified depression. The anxiety category included generalized anxiety disorder, social anxiety disorder, anxiety not otherwise specified, and unspecified anxiety. The category of suicidality included history of suicidal ideation, history of suicide attempt, or history of self-injurious behavior. This information was collected from the patient’s history and physical clinical note or from the psychological assessment during hospitalization.

Patient’s height and weight were used to calculate BMI [36]. Percent (%) mBMI was determined by dividing patient’s BMI at admission by the 50th percentile BMI for age using both female and male Centers for Disease Control and Prevention (CDC) BMI growth charts [37]. Treatment goal BMI for each patient at the University of California, San Francisco is determined using a patient’s historical growth trend and based on returning to a BMI percentile that predated onset of the eating disorder. Treatment goal BMI at corresponding BMI percentiles were plotted for each patient on CDC BMI growth charts. For the purposes of this manuscript, we use ‘birth-assigned sex growth chart’ to note plotting the patient’s anthropometric data on the growth chart consistent with their birth-assigned sex. For example, a transgender male’s birth-assigned sex growth chart would be plotting his growth data on the CDC female growth chart. We use ‘gender affirmed growth chart’ to note plotting the patient’s data on the growth chart consistent with their affirmed gender. For example, a transgender male’s gender affirmed growth chart would be plotting his growth data on the CDC male growth chart. For patients with a non-binary identity or ‘Other’ gender identity, we use birth-assigned sex growth chart to note plotting of data on the CDC female growth chart as all the non-binary patients were designated female at birth. We use ‘alternative growth chart’ to note plotting non-binary and gender fluid patient data on the CDC male growth chart. Laboratory results were coded as normal or abnormal based on University of California, San Francisco laboratory reference ranges.

Descriptive statistics were used to analyze data. Fisher’s exact, Chi square or *t*-tests were used to examine potential differences in medical, anthropometric, and psychiatric characteristics between gender diverse patients and cisgender patients. Analyses used Stata 17 (Stata Corp LP, College Station, TX).

## Results

Demographic, anthropometric, and medical characteristics of participants are shown in Table 1. Ten patients (2.2%) met criteria for this study. Of the 10 patients, 3 identified as transgender male, 3 as transgender female, 2 identified as non-binary, and 2 as other which included gender fluid and ‘no label’. The gender diverse patients were significantly younger compared to cisgender patients [13.6 (1.5) vs. 15.6 (2.7) years, *p* = 0.017] (Table 1). Gender diverse patients had a significantly higher % mBMI compared to cisgender peers [97.1 (14.8) vs. 87.9 (13.7)%, *p* = 0.037] (Table 1). Gender diverse patients did not differ from cisgender peers by average heart rate nadir or blood pressure nadir. Free thyroxine levels were lower in gender diverse versus cisgender patients [10.5 (3.5) vs. 12.0 (1.8) pmol/L, *p* = 0.008] (Table 1).

**Table 1:** Demographic, anthropometric, and medical characteristics of adolescents and young adults hospitalized for an eating disorder by gender identity

	Gender diverse^a^ (n = 10)	Cisgender (n = 453)	*P*-value^b^
Age, years	13.6 ± 1.5	15.6 ± 2.7	**0.017**
Race, n (%)			0.270
White or Caucasian	5 (50.0)	286 (63.1)	
Asian or NHOPI^c^	3 (30.0)	38 (6.4)	
Black or African American	0 (0.0)	14 (3.1)	
Other	2 (20.0)	97 (21.4)	
Unknown/Declined	0 (0.0)	18 (4.0)	
Ethnicity, n (%)			1.000
Hispanic	2 (20.0)	86 (19.0)	
Non-Hispanic	8 (80.0)	341 (75.3)	
Unknown/Declined	0 (0.0)	26 (5.7)	
BMI kg/m^2^	18.8 ± 2.9	17.8 ± 2.8	0.261
Percent median BMI^d^	97.1 ± 14.8	87.9 ± 13.7	**0.037**
Vital signs			
Pulse (beats per minute) nadir during hospitalization	44 ± 8.8	46 ± 10.6	0.501
Systolic pressure (mmHg) nadir during hospitalization	84 ± 7.1	84 ± 9.7	0.995
Diastolic pressure (mmHg) nadir during hospitalization	46 ± 5.8	45 ± 7.3	0.884
Electrolyte analysis at admission			
Sodium (mmol/L)	139 ± 1.6	138 ± 8.7	0.966
Potassium (mmol/L)	3.8 ± 0.3	3.9 ± 0.7	0.526
Magnesium (mg/dL)	2.1 ± 0.2	2.1 ± 0.2	0.882
Phosphorous (mg/dL)	3.9 ± 0.5	4.0 ± 2.1	0.892
Other laboratory evaluation at admission			
White blood cell count (× 10^9^/L)	5.4 ± 0.9	6.1 ± 1.8	0.222
Hemoglobin (g/dL)	12.8 ± 1.1	13.0 ± 1.2	0.698
Hematocrit (%)	38.0 ± 2.7	38.3 ± 3.2	0.776
Thyroid stimulating hormone (mIU/L)	1.5 ± 1.2	2.0 ± 1.4	0.274
Free thyroxine (pmol/L)	10.5 ± 3.5	12.0 ± 1.8	**0.008**
Aspartate transaminase (U/L)	25 ± 4.2	29 ± 35.4	0.725
Alanine transaminase (U/L)	20 ± 7.5	22 ± 29.1	0.849
Triglycerides (mg/dL)	25 ± 19.8	47 ± 33.3	0.111
Cholesterol (mg/dL)	163 ± 24.5	166 ± 41.6	0.849
Zinc (µg/dL)	64 ± 11.2	64 ± 14.7	0.950

Table values are mean ± SD for continuous variables and n (column %) for categorical variables

^a^Gender diverse refers to transgender, non-binary, gender fluid, and other

^b^*T*-test was used for continuous variables. Chi Square or Fisher's exact test if n < 5 was used for categorical variables

^c^NHOPI, Native Hawaiian and Other Pacific Islanders

^d^Patient’s BMI at admission divided by 50th percentile body mass index for age and sex

Bold indicates *P* < 0.05

Gender diverse patients had laboratory abnormalities as shown in Table 2. A significantly higher percentage of gender diverse patients had hypertriglyceridemia compared to cisgender patients (40 vs. 10.6%, *p* = 0.018) (Table 2). Hypercholesterolemia did not differ by group. Two (20%) of gender diverse patients had evidence of anemia, which did not differ from cisgender peers (*p* = 0.638) (Table 2). Electrolyte abnormalities were present in two (20%) gender diverse patients, with no differences as compared to cisgender peers (Table 2).

**Table 2:** Percentage of adolescents and young adults with laboratory abnormalities during entirety of hospitalization for an eating disorder by gender identity

	Gender diverse^a^ (n = 10)	Cisgender (n = 453)	*P*-value^b^
Hypokalemia (< 3.5 mmol/L)^c^	20.0 (2)	14.8 (67)	0.649
Hypomagnesemia (< 1.7 mg/dL)^c^	0.0 (0)	1.6 (7)	1.000
Hypophosphatemia (< 2.9 mg/dL)^c^	10.0 (1)	4.4 (20)	0.374
Leukopenia (< 4.5 × 10^9^/L)^c^	10.0 (1)	15.7 (71)	1.000
Anemia (< 11.8 g/dL)^c^	20.0 (2)	13.9 (63)	0.638
Elevated aspartate transaminase (> 35 U/L)^d^	20.0 (2)	15.5 (70)	0.659
Elevated alanine transaminase (> 24 U/L)^d^	50.0 (5)	26.3 (119)	0.141
Hypertriglyceridemia (> 129 mg/dL)^d^	40.0 (4)	10.6 (48)	**0.018**
Hypercholesterolemia (> 199 mg/dL)^d^	30.0 (3)	21.9 (99)	0.465

Table values are column percentage (n), percentages may not sum to 100% due to rounding

^a^Gender diverse refers to transgender, non-binary, gender fluid, and other

^b^Fisher’s exact test was used for categorical variables where n < 5

^c^Lower limit of normal values based on University of California San Francisco laboratory reference range

^d^Upper limit of normal values based on University of California San Francisco laboratory reference range

Bold indicates *P* < 0.05

Eating disorder diagnoses and psychiatric comorbidities are shown in Tables 3 and 4, respectively. A greater percentage of gender diverse patients had a diagnosis of OSFED, which were all atypical anorexia nervosa, compared to cisgender peers (70 vs. 31%, *p* = 0.009) (Table 3). There was no difference seen in the percent diagnosed with AN. Six (60%) gender diverse patients had a coexisting diagnosis of anxiety and 5 (50%) had a diagnosis of depression. Six (60%) gender diverse patients had more than one psychiatric illness at time of presentation. Four (40%) of gender diverse had a history of self-harm and/or suicidality. When compared to cisgender peers, a higher percent of gender diverse patients reported anxiety (60 vs. 28%, *p* = 0.037) (Table 4).

**Table 3:** Eating disorder diagnoses among adolescents and young adults hospitalized for an eating disorder by gender identity

	Gender diverse^a^ (n = 10)	Cisgender (n = 453)	*P*-value^b^
Anorexia nervosa^c^	30.0 (3)	58.7 (266)	0.103
Other specified feeding and eating disorder^d^	70.0 (7)	31.1 (141)	**0.009**
Other^e^	0.0 (0)	10.2 (46)	0.608

Table values are column percentage (n), percentages may not sum to 100% due to rounding

^a^Gender diverse refers to transgender, non-binary, gender fluid, and other

^b^Fisher’s exact test was used for categorical variables as n < 5

^c^Anorexia nervosa includes both restricting subtype and binge/purge subtype

^d^OSFED includes Atypical Anorexia Nervosa and Purging Disorder

^e^Other includes Avoidant Restrictive Food Intake Disorder, Bulimia nervosa, and unspecified eating disorder

Bold indicates *P* < 0.05

**Table 4:** Psychiatric comorbidities among adolescents and young adults hospitalized for an eating disorder by gender identity

	Gender diverse^a^ (n = 10)	Cisgender (n = 453)	*P*-value^b^
Depression^c^	40.0 (4)	30.0 (136)	0.499
History of suicidality^d^	40.0 (4)	21.9 (99)	0.240
Anxiety^e^	60.0 (6)	28.0 (127)	**0.037**

Table values are column percentage (n)

^a^Gender diverse refers to transgender, non-binary, gender fluid, and other

^b^Fisher’s exact test was used for categorical variables as n < 5

^c^Depression includes diagnoses of major depressive disorder, depressive disorder not otherwise specified, and unspecified depression

^d^History of suicidality includes history of suicide attempt, suicidal ideation or self-injurious behavior

^e^Anxiety includes diagnoses of generalized anxiety disorder, social anxiety disorder, anxiety not otherwise specified, and unspecified anxiety

Bold indicates *P* < 0.05

Comparisons of % mBMI evaluation using birth-assigned sex and gender-affirmed/alternative growth charts are shown for those with AN (Table 5) and OSFED (Table 6). A significant difference was seen in % mBMI between birth-assigned sex and gender affirmed/alternative growth charts for those gender diverse patients with a diagnosis of AN [85.5 (2.95) vs. 86.5 (2.88)%, *p* = 0.0031] (Table 5). For those with OSFED, the difference in % mBMI between growth charts followed a similar trend as was seen in AN, however the difference was not significant [106.7 (10.86) vs. 107.6 (11.47)%, *p* = 0.067]. As shown in Table 7, no difference was seen when comparing treatment goal BMI between birth-assigned sex and gender affirmed/alternative growth charts (Table 7).

**Table 5:** Comparison of percent median body mass index using birth-assigned sex and gender-affirmed/alternative growth charts in those with anorexia nervosa

Age	Sex assigned at birth	Gender identity	Percent mBMI based on sex assigned at birth growth curves	Percent mBMI based on gender-affirmed/alternative growth curves	*P*-value
					**0.0031**
12 years 6 months	Female	Non-binary	85.3	86.3	
13 years 5 months	Female	Other	84.1	85.5	
13 years 9 months	Female	Non-binary	89.6	90.5	
14 years 10 months	Female	Male	82.8	83.7	
		Average	85.5	86.5	

Bold indicates *P* < 0.05

**Table 6:** Comparison of percent median body mass index using birth-assigned sex and gender-affirmed/alternative growth charts in those with other specified feeding and eating disorder

Age	Sex assigned at birth	Gender identity	Percent mBMI based on sex assigned at birth growth curves	Percent mBMI based on gender-affirmed/alternative growth curves	*p*-value
					0.067
12 years 5 months	Female	Male	123.5	125.6	
12 years 8 months	Female	Other	108.6	110.4	
14 years 6 months	Female	Male	99.5	100	
14 years 10 months	Male	Female	100	99.5	
16 years 2 months	Male	Female	114	114.6	
16 years 5 months	Male	Female	94.3	95.2	
		Average	106.7	107.6

**Table 7:** Comparison of treatment goal body mass index using birth-assigned sex growth charts and gender-affirmed/alternative growth charts

Age	Sex assigned at birth	Gender identity	Treatment goal BMI based on sex assigned at birth growth curves	Treatment goal BMI based on gender-affirmed/alternative growth curves	*p*-value
					0.204
14 years 10 months	Female	Male	19.8	19.7	
12 years 6 months	Female	Non-binary	18.2	18.1	
13 years 9 months	Female	Non-binary	21.5	21.0	
14 years 6 months	Female	Male	22.0	21.6	
16 years 5 months	Male	Female	20.8	20.6	
13 years 5 months	Female	Other	18.9	18.7	
12 years 8 months	Female	Other	20.7	20.2	
16 years 2 months	Male	Female	29.0	27.6	
14 years 10 months	Male	Female	26.6	27.9	
12 years 5 months	Female	Male	23.3	22.4	
		Average	22.0	21.8	

## Discussion

To our knowledge this is one of the first studies of gender diverse adolescents admitted for medical stabilization from complications of eating disorders. Notably, the gender diverse patients were younger than cisgender peers and younger when compared to previous descriptive studies of inpatient samples [38–40]. These findings are worrisome as gender diverse adolescents with eating disorders are medically unstable at a younger age, suggesting that eating disorder behaviors could have started years prior or before adolescence. Previous evidence suggests that a younger onset of eating disorder behaviors leads to worsened outcomes [41, 42]. Thus, we recommend early screening for disordered eating/eating disorders among this population starting in childhood.

In addition to being younger, OSFED (specifically atypical anorexia nervosa), was the most common diagnosis among gender diverse patients. A greater percentage of gender diverse patients had a diagnosis of OSFED compared to cisgender peers. This corresponds to the gender diverse patients presenting for hospitalization with a higher % mBMI compared to cisgender peers. The findings seen with the gender diverse patients are consistent with what is known about atypical anorexia nervosa [43]. Gender diverse patients were just as medically unstable with bradycardia, hypotension, and laboratory abnormalities, despite a higher weight compared to cisgender peers. Gender diverse patients had a higher prevalence of hypertriglyceridemia compared to cisgender peers. The mechanism of hypertriglyceridemia in the gender diverse patients is unclear. We hypothesize that this could be related to malnutrition due to hepatic oxidative stress that has been described in animal studies [44]. Gender diverse patients had lower free thyroxine levels compared to cisgender peers which has been described as a potential endocrinologic change in those presenting with eating disorders at a younger age [41].

Determination of which growth charts to use for gender diverse youth with eating disorders remains a clinical conundrum [2, 29, 30]. Our study attempts to shed light on this question by evaluating our patients’ % mBMI at presentation and treatment goal BMI by using both birth-assigned sex growth charts and gender affirmed growth charts. While our results show a statistically significant difference between growth charts for % mBMI, this is not clinically significant in actual practice as patients remained in a similar category of malnutrition whether birth-assigned, or gender affirmed growth charts were used. We showed no difference in treatment goal BMI between growth charts. While we did not see statistical differences in treatment goal BMI between birth-assigned sex growth chart and gender affirmed growth chart, many suggest using the curves of the patient’s affirmed gender, particularly if the patient is on gender-affirming hormone therapy [7, 34, 35]. Thus, we conclude that the use of gender affirmed growth curves to plot % mBMI and treatment goal weight/BMI is a reasonable approach for gender diverse patients as there is minimal difference but will promote inclusive care. Using both standard male and female growth charts to determine average % mBMI and treatment goal BMI based on historical curves could be beneficial for non-binary and gender fluid patients as suggested in previous studies [2, 34, 35]. While our study does not show a difference between curves, a previous report by Kidd et al. showed differences at the extremes of growth curves using two theoretical cases [34]. Additionally, Lee et al. has shown differences in bone mineral density scores by comparing bone mineral density of gender diverse youth using male and female reference standards [45].

Of the 10 gender diverse patients, 3 were on gender affirming medical therapy at time of admission. These 3 patients all identified as female and were on feminizing therapy with estradiol and a GnRH agonist for testosterone suppression. Gender-affirming medical therapy can improve long term outcomes for transgender youth [35, 46, 47]; however, the patients in our study highlight that a patient can be on gender-affirming therapy and still struggle with an eating disorder. This suggests that providers should still screen for and recognize signs of eating disorders in gender diverse individuals regardless of affirming medical therapy.

A large percentage of our patients had psychiatric comorbidities as well as history of self-injurious behavior and suicidality. Of significance, gender diverse patients had more anxiety compared to cisgender peers. High rates of psychiatric comorbidities is a common theme among gender diverse adolescents, however, gender diverse adolescents with eating disorders are particularly vulnerable [14, 35].Given the psychiatric complexities of this population, we agree with Donaldson et al. who has previously suggested that interdisciplinary teams are vital in caring for gender diverse youth with eating disorders [35].

There are several limitations to this study that should be noted. First, the sample size of our gender diverse patient population is small and limited to 10 patients. This small sample size may limit the power to detect other significant differences from cisgender patients. The small sample size and given that this data was collected from a single tertiary care hospital in San Francisco, California may limit the generalizability of the findings to other inpatient populations. Second, this study is retrospective and observational nature, which precludes causal inferences. Additionally, a retrospective study encompassing 8 years brings up additional limitations, specifically for gender identity. Societal changes during 2012–2020 could have impacted providers inquiring about a patient’s gender identity, level of comfort a patient had in disclosing gender concerns, and access to gender affirming hormone therapy during these years. Another limitation includes the use of pre-existing psychiatric history and suicidality collected by self-report, introducing recall bias and heterogeneity into diagnostic reporting. Additionally, we were unable to fully evaluate the interplay that can be seen with body dysmorphia and development of eating disorders in gender diverse adolescents given the limited data available. As discussed previously, body dissatisfaction in gender diverse youth has been shown to be a risk factor for development of disordered eating, and we advocate for the evaluation of body dissatisfaction as part of eating disorder presentation in this population for future studies [1, 2, 5]. Additionally, we had limited data for the patients on gender-affirming hormone therapy and thus are not able to make conclusions about linear growth trends before and after starting therapy. Future areas of research should investigate the impact of gender-affirming hormone therapy on weight status and growth trends in this population.

Despite these limitations, to our knowledge we are the first to explore characteristics of gender diverse adolescents with eating disorders in an inpatient setting and to demonstrate that use of gender affirmed growth curves is a realistic approach with patient data.

## Conclusions

This study describes the medical, anthropometric, and psychiatric characteristics of transgender and other gender diverse adolescents with eating disorders admitted for medical stabilization and compares these characteristics to cisgender peers. We demonstrate that gender diverse patients present for medical stabilization at a younger age and higher weight compared to cisgender peers. Thus, it is imperative to screen these patients for disordered eating/eating disorder behaviors starting prior to adolescence with the goals of preventing medical instability and need for inpatient hospitalization. We show that differences in % mBMI between birth-assigned sex and gender affirmed growth curves can be detected. However, these differences in our sample were of minimal clinical significance, and thus we recommend referencing both growth curves to trend patients’ weight trajectory for gender diverse patients struggling with eating disorders. In particular, use of the gender affirmed/alternative growth charts in conjunction with birth-assigned sex charts can be a form of gender-affirming care for transgender, non-binary, and other gender diverse adolescents. Gender diverse patients demonstrate profound medical and psychiatric complications alongside their eating disorders. Larger prospective studies are needed to establish tailored, evidence-based treatment guidelines to improve long-term health outcomes for this vulnerable population.

## Data Availability

The data that support the findings of this study are available on request from the corresponding author, AC. The data are not publicly available due to confidentiality restrictions e.g., their containing information that could compromise the privacy of research participants.

## Abbreviations

% mBMI: Percent median body mass index
AN: Anorexia nervosa
BMI: Body mass index
CDC: Centers for Disease Control and Prevention
DSM-5: Diagnostic and Statistical Manual of Mental Disorders, Fifth Edition
DSM-IV: Diagnostic and Statistical Manual of Mental Disorders, Fourth Edition
GnRH: Gonadotropin-releasing hormone
OSFED: Other specified feeding and eating disorder
TGW: Treatment goal weight
